# Genome reduction as the dominant mode of evolution

**DOI:** 10.1002/bies.201300037

**Published:** 2013-06-25

**Authors:** Yuri I Wolf, Eugene V Koonin

**Affiliations:** National Center for Biotechnology Information, NLM, National Institutes of HealthBethesda, MA, USA

**Keywords:** ancestral reconstruction, archaea, genome complexification, genome reduction, horizontal gene transfer, orthologs

## Abstract

A common belief is that evolution generally proceeds towards greater complexity at both the organismal and the genomic level, numerous examples of reductive evolution of parasites and symbionts notwithstanding. However, recent evolutionary reconstructions challenge this notion. Two notable examples are the reconstruction of the complex archaeal ancestor and the intron-rich ancestor of eukaryotes. In both cases, evolution in most of the lineages was apparently dominated by extensive loss of genes and introns, respectively. These and many other cases of reductive evolution are consistent with a general model composed of two distinct evolutionary phases: the short, explosive, innovation phase that leads to an abrupt increase in genome complexity, followed by a much longer reductive phase, which encompasses either a neutral ratchet of genetic material loss or adaptive genome streamlining. Quantitatively, the evolution of genomes appears to be dominated by reduction and simplification, punctuated by episodes of complexification.

## Introduction: Complexity can either increase or decrease during the evolution of various life forms

The textbook depiction of the evolution of life on earth is that of an ascent toward a steadily increasing organismal complexity: from primitive protocells to prokaryotic cells to the eukaryotic cell to multicellular organisms to animals to humans, the crowning achievement of the entire history of life. On general grounds, this “progressivist” view of evolution has been repeatedly challenged, in particular in the eloquent writings of Gould 1. Gould argued that the average complexity of life forms has barely increased over the course of the history of life, even as the upper bound of complexity was being pushed upwards, perhaps for purely stochastic reasons, under a “drunkard's walk” model of evolution.

It has been well known for decades that the evolution of numerous parasitic and symbiotic organisms entails simplification rather than complexification. In particular, bacteria that evolve from free-living forms to obligate intracellular parasites can lose up to 95% of their gene repertoires without compromising the ancestral set of highly conserved genes involved in core cellular functions 2–3. The mitochondria, the ubiquitous energy-transforming organelles of eukaryotes, and the chloroplasts, the organelles responsible for the eukaryotic photosynthesis, are the ultimate realizations of bacterial reductive evolution 4–5. However, such reductive evolution, its paramount importance for eukaryotes notwithstanding, was considered to represent a highly specialized trend in the history of life.

From a more general standpoint, there are effectively irrefutable arguments for a genuine increase in complexity during evolution. Indeed, the successive emergence of higher grades of complexity throughout the history of life is impossible to ignore. Thus, unicellular eukaryotes that, regardless of the exact dating, evolved more than a billion years after the prokaryotes, obviously attained a new level of complexity, and multicellular eukaryotic forms, appearing even later, by far exceeded the complexity of the unicellular ones 5–8. Arguably, the most compelling is the argument from the origin of cellular life itself: before the first cells emerged, there must have been some much simpler (pre)biological replicating entities.

## Organismal complexity is hard to define but genomic complexity is much more tractable

Complexity is one of those all-important characteristics of any system that seems to be easily grasped intuitively (“we know it when we see it”) but is notoriously difficult to capture in a single, quantitative and constructive definition 9–10. The approach that comes the closest to meeting these criteria might involve the quantity known as Kolmogorov complexity (also known as algorithmic entropy), which is defined as the length of the shortest possible description of a system (often represented as a string of symbols) 11. However, Kolmogorov complexity is generally incomputable, and the concept is particularly difficult to apply to biological systems because of the non-trivial connection between the “description” (the genome) and the system itself (organismal phenotype). A useful practical approach to quantify the complexity of a system is to count the number of distinct parts of which it consists, and this is how organismal complexity is usually addressed by those that attempt to analyze it in a (semi) quantitative manner 12–13. Recently, McShea and Brandon 13 formulated the “First Law of Biology”, or the “Zero Force Law of Evolution” according to which unconstrained evolution leads to a monotonic increase in the average organismal complexity, due to purely the increase of entropy with time that is mandated by the second law of thermodynamics for any closed system.

However, the utility of equating complexity with entropy is dubious at best as becomes particularly clear when one attempts to define genomic complexity. Indeed, using sequence entropy (Shannon information) as a measure of genomic complexity is obviously disingenuous given that under this approach the most complex sequence is a truly random one that, almost by definition is devoid of any biological information. Hence, attempts have been made to derive a measure of biological complexity of a genome by equating it with the number of sites that are subject to evolutionary constraints, i.e. evolve under purifying selection 8,14. Although this definition of genomic complexity certainly is over-simplified, it shows intuitively reasonable trends, i.e. a general tendency to increase with organismal complexity 8. Moreover, introducing the additional definition of biological information density, that is per-site complexity, one can, at least in principle, describe distinct trends in genome evolution such as a trend toward high information density that is common in prokaryotes and the contrasting trend toward high complexity at low density that is typical in multicellular organisms 8. At a coarse-grain level, biological complexity of a genome can be redefined as the number of genes that are conserved at a defined evolutionary distance. Unlike the number of sites that are subject to selection, the conserved genes are rather easy to count, so this quantity became the basis for many reconstructions of genome evolution 16–17.

The relationship between genomic complexity and the complexity at various levels of the phenotype, from molecular to organismal, is far from being straightforward as it has become clear already in the pre-genomic era 18. Comparative genomics reinforced the complex relationships between the different levels of complexity in the most convincing manner by demonstrating the lack of a simple link between genomic and organismal complexities 19. Suffice it to note that the largest bacterial genomes encompass almost as many genes as some “obviously” complex animals, such as for example flies, and more than many fungi. One of the implications of these comparisons is that there could be other measures of genomic complexity that might complement the number of conserved genes and perhaps provide a better proxy for organismal complexity. For example, in eukaryotes, a candidate for such a quantity could be the intron density that reflects the potential for alternative splicing 20.

Genomic complexity is far easier to quantify than phenotypic complexity (even if the latter is easier to recognize intuitively). Indeed, the remarkable progress of genome sequencing, combined with the development of computational methods for advanced comparative genomics, provides for increasingly reliable reconstruction of ancestral genomes which transforms the study of the evolution of complexity from being a speculative exercise to becoming an evidence-based research direction. Here, we examine the results of such reconstructions and make an argument that reductive evolution resulting in genome simplification is the quantitatively dominant mode of evolution.

## Genome reduction pervades evolution

A reconstruction of genome evolution requires that the genes from the analyzed set of genomes are clustered into orthologous sets that are then used to extract patterns of gene presence-absence in the analyzed species. The patterns are superimposed on the evolutionary tree of these species and the gene compositions of the ancestral forms as well as the gene losses and gain along the tree branches are reconstructed using either maximum parsimony (MP) or maximum likelihood (ML) methods (see Box 1) 21–24. The ML methods yield much more robust reconstructions than the MP methods but also require more data. Similar methods can be applied to reconstruct evolution of other features for which orthologous relationships can be established, e.g. intron positions in eukaryotic genes.

## Box 1 Reconstruction of ancestral genomes: Maximum parsimony and maximum likelihood approaches

**Dollo Parsimony.** Only one gain per character is allowed; the pattern of losses, sufficient to produce the observed presence-absence pattern, with the minimum number of losses, is selected 86–87.

**Weighted Parsimony.** The relative gain-to-loss weight is set prior to reconstruction; the pattern of losses and gains with the minimum weighted score, sufficient to produce the observed presence-absence pattern, is selected 88,89.

**Maximum Likelihood.** Gain and loss probabilities per unit of time (possibly different for different tree branches) are the parameters; the presence-absence pattern and tree branch lengths are observed; the set of parameters and the gain-loss pattern, maximizing the likelihood of the observed presence-absence pattern, is selected 21–24.

**Figure:**
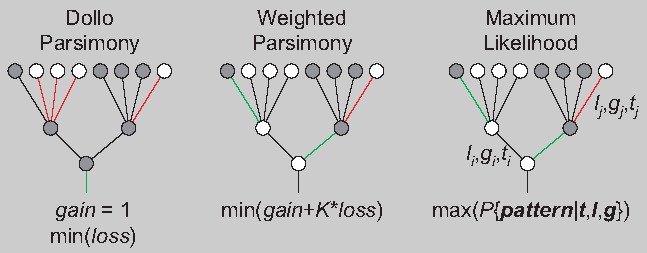


The figure illustrates the analysis of a pattern for a single character. Red lines indicate branches with losses; green lines indicate branches with gains.

Certainly, we are far from being able to obtain comprehensive evolutionary reconstructions for all or even most life forms. Nevertheless, reconstructed evolutionary scenarios are accumulating, some of them covering wide phylogenetic spans, and many of these reconstructions point to genome reduction as a major evolutionary trend (Table 1). The most dramatic but also the most obvious are the evolutionary scenarios for intracellular parasitic and symbiotic bacteria that have evolved from numerous groups of free-living ancestors. A typical example is the reductive evolution of the species of the intracellular parasites *Rickettsia* from the ancestral “Mother of *Rickettsia*” 25–26. Reductive evolution of endosymbionts can yield bacteria with tiny genomes consisting of 150–200 genes and lacking some essential genes such as those encoding several aminoacyl-tRNA synthetases, which is suggestive of an ongoing transition to an organelle state 3. Indeed, the ultimate cases of reductive evolution involve the mitochondria and chloroplasts that have lost nearly all ancestral genes (e.g. 13 out of the several thousand genes in the ancestral alpha-proteobacterial genome are retained in animal mitochondria) or literally all genes in the case of hydrogenosomes and mitosomes 27. Certainly, in this case, the evolutionary scenario appears as ultimate reduction “from the point of view” of the symbiont; the complexity of the emerging chimeric organism drastically increases, both at the genomic and at the phenotypic level, and it has been argued that such complexification would not have been attainable if not for the endosymbiosis 5–28. Furthermore, hundreds of genes, in the case of the mitochondrion, and even thousands in the case of the chloroplast, were not lost but rather transferred from the endosymbiont genome to the nuclear genome of the host 29,30.

**Table 1:** Reconstructions of the genome evolution for major groups of prokaryotes and eukaryotes

Taxa	Depth of evolutionary reconstruction	Subject of evolutionary reconstruction	Outcome	Reference
Mitochondria	Proto-mitochondrial (alpha-proteobacterial) endosymbiosis, presumably, last common ancestor of eukaryotes	Genes	Deep reduction, to the point of genome elimination in anaerobic protists containing hydrogenosomes or mitosomes.	4–5
Lactobacillales	Last common ancestor of bacilli	Gene Families	Complex ancestor; dominance of the reduction mode in all lineages	34–35
*Anoxybacillus flavithermus*	Last common ancestor of Firmicutes	Gene families	Ancestral complexification, then reduction	85
Rickettsia	Last common ancestor (“mother”) of rickettsia	Genes	Complex ancestor, dominance of the reduction mode in all lineages	25–26
Cyanobacteria including chloroplasts	Last common ancestor of cyanobacteria	Genes	Complex ancestor, complexification in some lineages, reduction in other lineages, ultimate reduction in chloroplasts	41
Archaea	Last archaeal common ancestor	Gene families	Moderately complex ancestor, ancestral complexification in some lineages, more recent dominance of genome reduction in all lineages	42
Eukaryotes	Last eukaryotic common ancestor	Protein domain families	Complex ancestor, reduction of the domain repertoire in most lineages, expansion only in multicellular organisms	51
Eukaryotes	Last common ancestor of eukaryotes	Introns	Complex early ancestors, mostly reductive evolution, complexification in some, primarily multicellular lineages	20–53
Microsporidia	Last common ancestor of microsporidia	Genes	Complex ancestor, deep reduction	32

Deep genome reduction, with the smallest sequenced genome of only 2.9 Mb, is also observed in *Microsporidia*, the eukaryotic intracellular parasites that appear to be highly derived fungi 32. The most extreme genome reduction among eukaryotes is observed in nucleomorphs which are remnants of algal endosymbionts present in cryptophytes and chlorarachniophytes and retain only a few hundred genes 33.

Beyond parasites and symbionts, reductive evolution was observed in several groups of organisms that evolved a commensal life style. One of the best-characterized cases involves the *Lactobacillales*, a group of Gram-positive bacteria that is extremely common in a variety of animal- and plant-associated habitats. A maximum parsimony reconstruction revealed substantial gene loss, from ∼3,000 genes in the common ancestor of *Bacilli* to ∼1,300–1,800 genes in various *Lactobacilli* species 34–35. The genes apparently have been lost in a stepwise manner, with substantial loss associated with each internal branch of the tree and most but not all of the individual species. These losses were only to a small extent offset by inferred gain of new genes.

Certainly, the evolution of the genomes of parasites, symbionts and commensals is not a one-way path of reduction. On the contrary, the reduction ratchet is constrained by the advantages of retaining certain metabolic pathways that complement the host metabolism 36–37. Notably, mathematical modeling of the evolution of the insect endosymbiont *Buchnera aphidicola* showed that metabolic requirements could determine not only the end point of genomic reduction but to some extent also the order of the gene deletion 38. Moreover, the reductive trend is countered by proliferation of genes involved in parasite-host interaction such as, for example, ankyrin repeat proteins that act as secreted virulence factors 39–40. Quantitatively, however, in most parasites and symbionts, these processes make a relatively minor contribution compared to the massive genome reduction.

An evolutionary reconstruction for *Cyanobacteria*, an expansive bacterial phylum that consists mostly of free-living forms and includes some of the most complex prokaryotes, produced mixed results, with several lineages characterized by genome expansion 41. Nevertheless, even in these organisms, evolution of one of the two major branches was dominated by extensive genes loss, and several lineages were mostly losing genes in the other major branch.

**Figure 1:**
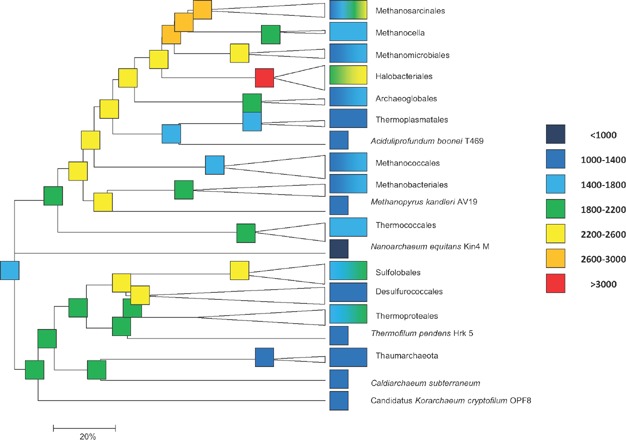
Reconstruction of the evolution of the archaea.The color code indicates the number of genes that belongs to clusters of archaeal orthologous genes (arCOGs) in the extant genomes and the reconstructed number of arCOGs in the ancestral forms 42. The figure is modified from 42.

Conceivably, the most compelling evidence of the dominance of genome reduction and simplification was obtained through the reconstruction of the genomic evolution of archaea that almost exclusively are free-living organisms 17–42. The latest ML reconstruction based on a comparative analysis of 120 archaeal genomes traced between 1,400 and 1,800 gene families to the last common ancestor of the extant archaea 42. Given the fractions of conserved and lineage-specific genes in modern archaeal genomes, this translates into approximately 2,500 genes in the ancestral genome, which is a larger genome than most of the extant archaea possess (Fig. 1). The reconstructed pattern of gene loss and gain in archaea is non-trivial: there seems to have been some net gene gain at the base of each of the major archaeal branches that was almost invariably followed by substantial gene loss; as discussed below, this could be a general pattern of genome evolution. The notable exceptions are *Halobacteria* and *Methanosarcinales*, the two archaeal lineages in which evolution was strongly impacted by horizontal gene transfer from bacteria 43–44 that offset the gene loss and led to genome expansion (Fig. 1). Although less reliable than the genome-wide ML reconstructions, attempts on the reconstruction of the ancestral state of specific functional systems seem to imply even more striking complexity of archaeal ancestors. For example, comparative analysis of the cell divisions machineries indicates that the common ancestor of the extant archaea might have possessed all three varieties of the division systems found in modern forms 45.

Reconstructions of the evolution of eukaryotic genomes yielded expanding ancestors as the number of diverse genomes available for comparative analysis grew. At least until recently, the available collection of eukaryotic genomes remained insufficient for reliable ML reconstruction. However, maximum parsimony reconstruction traced between 4,000 and 5,000 to the last eukaryotic common ancestor (LECA) 46–47. An even simpler analysis identified over 4,000 genes that are shared between *Naegleria gruberi*, the first free-living excavate (one of the supergroups of unicellular eukaryotes that also includes parasitic forms such as trichomonas and giardia) for which the genome was sequenced and at least one other supergroup of eukaryotes, suggesting that these genes were inherited from the LECA 48–49. Such estimates are highly conservative as they disregard parallel gene loss in different major lineages, an important phenomenon in the evolution of eukaryotes. Indeed, even animals and plants, the eukaryotic kingdoms that seem to be the least prone to gene loss, have lost about 20% of the putative ancestral genes identified in the unicellular *Naegleria*. Collectively, these findings imply that the genome of the LECA was at least as complex as the genomes of typical extant free-living unicellular eukaryotes 50. Even more striking conclusions were reached by the reconstruction of the evolution of the eukaryotic protein domain repertoire that involved comparison of 114 genomes 51. The results of this reconstruction indicate that most of the major eukaryotic lineages have experienced a net loss of domains that have been traced to the LECA. Substantial increase in protein complexity appears to be associated only with the onset of the evolution of the two kingdoms of multicellular eukaryotic organisms, plants, and animals.

Remarkably congruent results have been obtained in reconstructions of the gain and loss of introns in eukaryotic genes. In this case, the availability of thousands intron positions provide for the use of powerful ML methods. The reconstructions consistently indicate that ancestral eukaryotes including the LECA and the founders of each supergroup were intron-rich forms, with intron densities higher than those in the genes of most extant eukaryotes and probably only slightly lower than those in the modern organisms with the most complex gene structures, such as mammals 20,52. Remarkably, intron-rich ancestors were reconstructed even for those major groups of eukaryotes that currently consist entirely of intron-poor forms such as the alveolates that apparently evolved via differential, lineage-specific, extensive intron loss 54. All in all, intron loss clearly dominated the evolution of eukaryotic genes, with episodes of substantial gain linked only with the emergence of some major groups, especially animals 20–53, in full agreement with the results of the evolutionary reconstruction for the eukaryotic domain repertoire 51. As previously pointed out by Brinkmann and Philippe 55, simplification could be an “an equal partner to complexification” in the evolution of eukaryotes. The latest reconstruction results suggest that simplification could be even “more equal” than complexification.

## Both neutral and adaptive routes lead to genome reduction

Genome reduction in different life forms seems to have occurred via two distinct routes: (i) the neutral gene loss ratchet and (ii) adaptive genome streamlining 8–56. Typically, the reductive evolution of intracellular pathogens does not seem to be adaptive inasmuch as the gene loss does not appear to occur in parallel with other trends suggestive of streamlining such as shrinking of intergenic regions or intense selection on protein-coding sequences manifest in a low *Ka*/*Ks* ratio. On the contrary, the intracellular bacteria appear to rapidly evolve under weak selection 3–57. The lack of correlation between different genomic features that are generally viewed as hallmarks of adaptive genome streamlining (i.e. selection for rapid replication), along with the presence of numerous pseudogenes that seem to persist for relatively long time spans and similarly persistent mobile elements 3–60, implies that in these organisms genomic reduction stems from neutral ratchet-like loss of genes that are non-essential for intracellular bacteria. This route of evolution conceivably was enabled by the virtual sequestration of intracellular parasites and symbionts from HGT and by the ensuing reduction of the effective population size 61,62. This apparent non-selective mode of gene loss is compatible with the small effective population size of parasites and symbionts, which results in an increased evolutionary role of genetic drift and infeasibility of strong selection 64–65. On a long-term evolutionary scale, these organisms are likely to be headed for extinction due to the diminished evolutionary flexibility that reduces their chance of survival in case of environment change 66. Coming back to the definitions introduced above, in the evolution of parasites and symbionts, the decrease in the biological complexity of genomes occurs in parallel with the decrease in information density.

However, bona fide adaptive genome streamlining appears to be a reality of evolution as well. Features of such streamlining are detectable in the genomes of the highly successful free-living organisms such as the cyanobacterium *Prochlorococcus sp*. 67–68 and the alpha-proteobacteriumCandidatus *Pelagibacter ubique*, apparently the most abundant cellular life forms on earth 56,69. These bacteria possess highly compact genomes and evolve under strong purifying selection suggesting that in these cases, the loss of non-essential genes, mobile elements and intergenic regions is indeed driven by powerful selection for rapid genome replication and minimization of the resources required for growth. Genome evolution of these highly successful life forms involves a drop in the overall complexity but an increase in information density. Of course, all the pressure of genome streamlining notwithstanding, the lifestyle of these free-living, autotrophic organisms imposes non-negotiable constraints on the extent of gene loss in these organisms because they have to maintain complete, even if minimally diversified metabolic networks. Additionally, an important factor in the evolution of these organisms that dwell in microbial communities could the “Black Queen effect” whereby selection operates at the community level so that otherwise essential genes can be lost as long as the respective metabolites or other commodities are provided to some community members 56–71.

Reconstructions of genome evolution in both prokaryotes and eukaryotes indicate that the loss of genes and introns typically occurs roughly proportionally to time, thus conforming with a form of genomic molecular clock 53–75. In contrast, the gain of genes and introns appears to be sporadic and mostly associated with major evolutionary innovations, such as in particular the origin of animals and plants. Thus, it has been concluded that gene loss is mostly neutral, within the constraints imposed by gene-specific purifying selection, whereas gene gain is controlled by positive selection 75. The former conclusion seems to be robust whereas the latter is dubious as gene gain in transitional epochs could be more plausibly attributed to genetic drift enabled by the population bottlenecks that are characteristic of these turbulent periods of evolution 8,65.

In cases of both neutral and adaptive genome reduction, this process appears to involve specialization contingent on environmental predictability whereas the bursts of innovation considerably opens up multiple new niches for exploration by evolving organisms.

## A biphasic model of evolution

**Figure 2:**
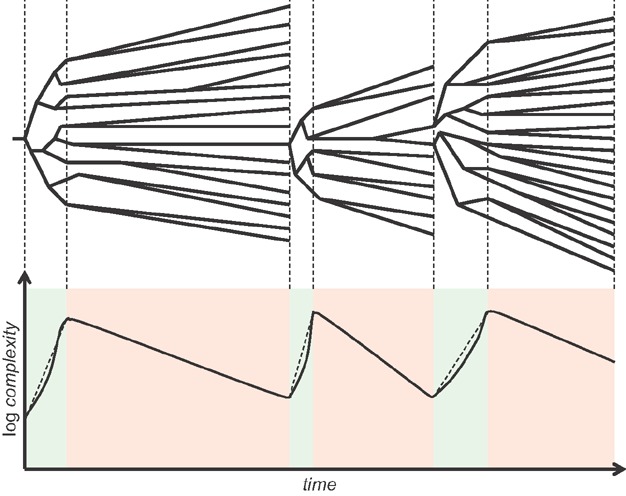
The biphasic model of punctuated evolution of genomes. Top: Periods of compressed cladogenesis punctuating long phases of quasi-stasis in the history of a particular lineage. Bottom: Complexity profile. The vertical axis implies the biological complexity of genomes that can be expressed as the number of sites or genes that are subject to selection. The green background indicates the complexification phase and the red background indicates the reduction phase.The dashed lines indicate the super-exponential growth rate in the complexification phase.

The findings that in many if not most lineages evolution is dominated by gene (and more generally, DNA) loss that occurs in a roughly clock-like manner whereas gene gain occurs in bursts associated with the emergence of major new groups of organisms imply a biphasic model of evolution (Fig. 2). Under this model, the evolutionary process in general can be partitioned into two phases of unequal duration: (i) genomic complexification at faster than exponential rate that is associated with stages of major innovation and involves extensive gene duplication, gene gain from various sources, in particular horizontal gene transfer including that from endosymbionts, and other genomic embellishments such as eukaryotic introns, and (ii) genomic simplification associated with the gradual loss of genes and genetic material in general, typically at the rate of exponential decay. The succession of the two phases appears to be a recurrent pattern that defines the entire course of the evolution of life. The first, innovative phase of evolution is temporally brief, engenders dramatic genomic and phenotypic perturbations, and is linked to population bottlenecks. The second, reductive phase that represents “evolution as usual” is protracted in time, is facilitated by the deletion bias that seems to be a general feature of genome evolution 77,78, and is associated either with a continuously small effective population size, as in parasites and symbionts with decaying genomes, or with evolutionary success and increasing effective population size as in free-living organisms undergoing genome streamlining 56,57. Clearly, the reductive phase of evolution is not limited to the loss of genes that were acquired in a preceding burst of innovation. An excellent case in point is the evolution of eukaryotes, where the explosive phase of eukaryogenesis yielded duplications of a substantial number of genes. Many of these gene duplicates diversified and persisted throughout the course of eukaryote evolution whereas numerous other genes were lost in multiple lineages 46,47.

Interestingly, detailed reconstruction of the independent processes of reductive evolution in several parasitic bacteria appears to reveal a “domino effect” that, on a much smaller evolutionary scale, causes punctuation in reductive evolution itself 80. It appears that the gradual, stochastic course of gene death is punctuated by occasional bursts when a gene belonging to a functional module or pathway is eliminated, rendering useless the remaining genes in the same module or pathway.

Certainly, the biphasic model of evolution depicted in Fig. 2 is not all-encompassing as continuous, long-term increase in genome complexity (but not necessarily biological information density) is observed in various lineages, our own history (that is, evolution of vertebrates) being an excellent case in point. Nevertheless, to the best of our present understanding informed by the reconstructions of genome evolution, extensive loss of genetic material punctuated by bursts of gain is the prevailing mode of evolution.

The biphasic model of evolution presented here expands on the previously developed scenario of compressed cladogenesis 81,82. It also conceptually reverberates with Gould's and Eldredge's punctuated equilibrium model 84, where the periods of “stasis” actually represent relatively slow genome dynamics that in many if not most lines of descent is dominated by the loss of genetic material.

## Conclusions and outlook

The results of evolutionary reconstructions for highly diverse organisms and through a wide range of phylogenetic depths indicate that contrary to widespread and perhaps intuitively plausible opinion, genome reduction is a dominant mode of evolution that is more common than genome complexification, at least with respect to the time allotted to these two evolutionary regimes. In other words, many if not most major evolving lineages appear to spend much more time in the reductive mode than in the complexification mode. The two regimes seem to differ also qualitatively in that genome reduction seems to occur more or less gradually, in a roughly clock-like manner, whereas genome complexification appears to occur in bursts accompanying evolutionary transitions. Genome reduction apparently occurs in two distinct and distinguishable manners, i.e. either via a neutral ratchet of genetic material loss or by adaptive genome streamlining.

Despite the diversity of the available case stories of reductive evolution, the current material is obviously insufficient for an accurate estimation of the relative contributions of genome reduction and complexification to the evolution of different groups of organisms. To derive such estimates, evolutionary reconstructions on dense collections of genomes from numerous taxa are required. Even more detailed analysis including careful mapping of loss and gain of genetic material to specific stages of evolution is necessary to refute or validate the model of punctuated genome evolution outlined here. In a more abstract plane, a major goal for future work is the development of a rigorous theory to explain biphasic evolution with the populations' dynamic framework.

## Abbreviations

LECA: last eukaryotic common ancestor
ML: maximum likelihood
MP: maximum parsimony

## References

[b1] Gould SJ (1997). Full House: The Spread of excellence from Plato to Darwin.

[b2] Moran NA (2002). Microbial minimalism: genome reduction in bacterial pathogens. Cell.

[b3] McCutcheon JP, Moran NA (2012). Extreme genome reduction in symbiotic bacteria. Nat Rev Microbiol.

[b4] Embley TM, Martin W (2006). Eukaryotic evolution, changes and challenges. Nature.

[b5] Lane N, Martin W (2010). The energetics of genome complexity. Nature.

[b6] Lane N (2011). Energetics and genetics across the prokaryote-eukaryote divide. Biol Direct.

[b7] Lynch M (2007). The Origins of Genome Archiecture.

[b8] Koonin EV (2011). The Logic of Chance: The Nature and Origin of Biological Evolution.

[b9] Gell-Mann M (1995). The Quark and the Jaguar: Adventures in the Simple and the Complex.

[b10] Johnson N (2009). Simply Complexity: A Clear Guide to Complexity Theory.

[b11] Li M, Vitanyi PMB (2008). An Introduction to Kolmogorov Complexity and its Applications.

[b12] McShea D (2000). Functional complexity in organisms: parts as proxies. Biol Philos.

[b13] McShea DW, Brandon R (2010). Biology's First Law.

[b14] Adami C (2002). What is complexity. BioEssays.

[b15] Koonin EV (2004). A non-adaptationist perspective on evolution of genomic complexity or the continued dethroning of man. Cell Cycle.

[b16] Ouzounis CA (2005). Ancestral state reconstructions for genomes. Curr Opin Genet Dev.

[b17] Csuros M, Miklos I (2009). Streamlining and large ancestral genomes in archaea inferred with a phylogenetic birth-and-death model. Mol Biol Evol.

[b18] Lewontin RC (1974). The Genetic Basis of Evolutionary Change.

[b19] Koonin EV (2009). Evolution of genome architecture. Int J Biochem Cell Biol.

[b20] Rogozin IB, Carmel L, Csuros M, Koonin EV (2012). Origin and evolution of spliceosomal introns. Biol Direct.

[b21] Bollback JP (2006). SIMMAP: stochastic character mapping of discrete traits on phylogenies. BMC Bioinform.

[b22] Csuros M (2010). Count: evolutionary analysis of phylogenetic profiles with parsimony and likelihood. Bioinformatics.

[b23] Cohen O, Rubinstein ND, Stern A, Gophna U (2008). A likelihood framework to analyse phyletic patterns. Philos Trans R Soc Lond B Biol Sci.

[b24] Cohen O, Pupko T (2011). Inference of gain and loss events from phyletic patterns using stochastic mapping and maximum parsimony-a simulation study. Genome Biol Evol.

[b25] Blanc G, Ogata H, Robert C, Audic S (2007). Reductive genome evolution from the mother of *Rickettsia*. PLoS Genet.

[b26] Merhej V, Raoult D (2011). Rickettsial evolution in the light of comparative genomics. Biol Rev Camb Philos Soc.

[b27] Hjort K, Goldberg AV, Tsaousis AD, Hirt RP (2010). Diversity and reductive evolution of mitochondria among microbial eukaryotes. Philos Trans R Soc Lond B Biol Sci.

[b28] Martin W, Koonin EV (2006). Introns and the origin of nucleus-cytosol compartmentation. Nature.

[b29] Gabaldon T, Huynen MA (2005). Lineage-specific gene loss following mitochondrial endosymbiosis and its potential for function prediction in eukaryotes. Bioinformatics.

[b30] Gabaldon T, Huynen MA (2007). From endosymbiont to host-controlled organelle: the hijacking of mitochondrial protein synthesis and metabolism. PLoS Comput Biol.

[b31] Timmis JN, Ayliffe MA, Huang CY, Martin W (2004). Endosymbiotic gene transfer: organelle genomes forge eukaryotic chromosomes. Nat Rev Genet.

[b32] Corradi N, Slamovits CH (2011). The intriguing nature of microsporidian genomes. Brief Funct Genomics.

[b33] Moore CE, Archibald JM (2009). Nucleomorph genomes. Annu Rev Genet.

[b34] Makarova K, Slesarev A, Wolf Y, Sorokin A (2006). Comparative genomics of the lactic acid bacteria. Proc Natl Acad Sci USA.

[b35] Makarova KS, Koonin EV (2007). Evolutionary genomics of lactic acid bacteria. J Bacteriol.

[b36] Moran NA, McCutcheon JP, Nakabachi A (2008). Genomics and evolution of heritable bacterial symbionts. Annu Rev Genet.

[b37] Zaneveld J, Turnbaugh PJ, Lozupone C, Ley RE (2008). Host-bacterial coevolution and the search for new drug targets. Curr Opin Chem Biol.

[b38] Yizhak K, Tuller T, Papp B, Ruppin E (2011). Metabolic modeling of endosymbiont genome reduction on a temporal scale. Mol Syst Biol.

[b39] Al-Khodor S, Price CT, Kalia A, Abu Kwaik Y (2010). Functional diversity of ankyrin repeats in microbial proteins. Trends Microbiol.

[b40] Dubreuil R, Segev N (2011). Bringing host-cell takeover by pathogenic bacteria to center stage. Cell Logist.

[b41] Larsson J, Nylander JA, Bergman B (2011). Genome fluctuations in cyanobacteria reflect evolutionary, developmental and adaptive traits. BMC Evol Biol.

[b42] Wolf YI, Makarova KS, Yutin N, Koonin EV (2012). Updated clusters of orthologous genes for archaea: a complex ancestor of the archaea and the byways of horizontal gene transfer. Biol Direct.

[b43] Deppenmeier U, Johann A, Hartsch T, Merkl R (2002). The genome of *Methanosarcina mazei*: evidence for lateral gene transfer between bacteria and archaea. J Mol Microbiol Biotechnol.

[b44] Rhodes ME, Spear JR, Oren A, House CH (2011). Differences in lateral gene transfer in hypersaline versus thermal environments. BMC Evol Biol.

[b45] Makarova KS, Yutin N, Bell SD, Koonin EV (2010). Evolution of diverse cell division and vesicle formation systems in archaea. Nat Rev Microbiol.

[b46] Koonin EV, Fedorova ND, Jackson JD, Jacobs AR (2004). A comprehensive evolutionary classification of proteins encoded in complete eukaryotic genomes. Genome Biol.

[b47] Makarova KS, Wolf YI, Mekhedov SL, Mirkin BG (2005). Ancestral paralogs and pseudoparalogs and their role in the emergence of the eukaryotic cell. Nucleic Acids Res.

[b48] Fritz-Laylin LK, Prochnik SE, Ginger ML, Dacks JB (2010). The genome of *Naegleria gruberi* illuminates early eukaryotic versatility. Cell.

[b49] Koonin EV (2010). Preview. the incredible expanding ancestor of eukaryotes. Cell.

[b50] Koonin EV (2010). The origin and early evolution of eukaryotes in the light of phylogenomics. Genome Biol.

[b51] Zmasek CM, Godzik A (2011). Strong functional patterns in the evolution of eukaryotic genomes revealed by the reconstruction of ancestral protein domain repertoires. Genome Biol.

[b52] Roy SW (2006). Intron-rich ancestors. Trends Genet.

[b53] Csuros M, Rogozin IB, Koonin EV (2011). A detailed history of intron-rich eukaryotic ancestors inferred from a global survey of 100 complete genomes. PLoS Comput Biol.

[b54] Csuros M, Rogozin IB, Koonin EV (2008). Extremely intron-rich genes in the alveolate ancestors inferred with a flexible maximum-likelihood approach. Mol Biol Evol.

[b55] Brinkmann H, Philippe H (2007). The diversity of eukaryotes and the root of the eukaryotic tree. Adv Exp Med Biol.

[b56] Morris JJ, Lenski RE, Zinser ER (2012). The Black Queen Hypothesis: evolution of dependencies through adaptive gene loss. MBio.

[b57] Novichkov PS, Wolf YI, Dubchak I, Koonin EV (2009). Trends in prokaryotic evolution revealed by comparison of closely related bacterial and archaeal genomes. J Bacteriol.

[b58] Wernegreen JJ (2005). For better or worse: genomic consequences of intracellular mutualism and parasitism. Curr Opin Genet Dev.

[b59] Burke GR, Moran NA (2011). Massive genomic decay in *Serratia symbiotica*, a recently evolved symbiont of aphids. Genome Biol Evol.

[b60] Degnan PH, Ochman H, Moran NA (2011). Sequence conservation and functional constraint on intergenic spacers in reduced genomes of the obligate symbiont *Buchnera*. PLoS Genet.

[b61] Mamirova L, Popadin K, Gelfand MS (2007). Purifying selection in mitochondria, free-living and obligate intracellular proteobacteria. BMC Evol Biol.

[b62] O'Fallon B (2008). Population structure, levels of selection, and the evolution of intracellular symbionts. Evolution.

[b63] Ferrari J, Vavre F (2011). Bacterial symbionts in insects or the story of communities affecting communities. Philos Trans R Soc Lond B Biol Sci.

[b64] Lynch M (2006). Streamlining and simplification of microbial genome architecture. Annu Rev Microbiol.

[b65] Lynch M, Conery JS (2003). The origins of genome complexity. Science.

[b66] Merhej V, Raoult D (2012). Rhizome of life, catastrophes, sequence exchanges, gene creations, and giant viruses: how microbial genomics challenges Darwin. Front Cell Infect Microbiol.

[b67] Dufresne A, Garczarek L, Partensky F (2005). Accelerated evolution associated with genome reduction in a free-living prokaryote. Genome Biol.

[b68] Partensky F, Garczarek L (2010). Prochlorococcus: advantages and limits of minimalism. Ann Rev Mar Sci.

[b69] Giovannoni SJ, Tripp HJ, Givan S, Podar M (2005). Genome streamlining in a cosmopolitan oceanic bacterium. Science.

[b70] Viklund J, Ettema TJ, Andersson SG (2012). Independent genome reduction and phylogenetic reclassification of the oceanic SAR11 clade. Mol Biol Evol.

[b71] Sachs JL, Hollowell AC (2012). The origins of cooperative bacterial communities. MBio.

[b72] Carmel L, Rogozin IB, Wolf YI, Koonin EV (2007). Patterns of intron gain and conservation in eukaryotic genes. BMC Evol Biol.

[b73] Carmel L, Wolf YI, Rogozin IB, Koonin EV (2007). Three distinct modes of intron dynamics in the evolution of eukaryotes. Genome Res.

[b74] Novichkov PS, Omelchenko MV, Gelfand MS, Mironov AA (2004). Genome-wide molecular clock and horizontal gene transfer in bacterial evolution. J Bacteriol.

[b75] Snel B, Bork P, Huynen MA (2002). Genomes in flux: the evolution of archaeal and proteobacterial gene content. Genome Res.

[b76] Lynch M (2007). The frailty of adaptive hypotheses for the origins of organismal complexity. Proc Natl Acad Sci USA.

[b77] Petrov DA (2002). DNA loss and evolution of genome size in *Drosophila*. Genetica.

[b78] Gregory TR (2004). Insertion-deletion biases and the evolution of genome size. Gene.

[b79] Kuo CH, Ochman H (2009). Deletional bias across the three domains of life. Genome Biol Evol.

[b80] Dagan T, Blekhman R, Graur D (2006). The “domino theory” of gene death: gradual and mass gene extinction events in three lineages of obligate symbiotic bacterial pathogens. Mol Biol Evol.

[b81] Rokas A, Carroll SB (2006). Bushes in the tree of life. PLoS Biol.

[b82] Rokas A, Kruger D, Carroll SB (2005). Animal evolution and the molecular signature of radiations compressed in time. Science.

[b83] Puigbo P, Wolf YI, Koonin EV (2009). Search for a tree of life in the thicket of the phylogenetic forest. J Biol.

[b84] Gould SJ, Eldredge N (1993). Punctuated equilibrium comes of age. Nature.

[b85] Saw JH, Mountain BW, Feng L, Omelchenko MV (2008). Encapsulated in silica: genome, proteome and physiology of the thermophilic bacterium *Anoxybacillus flavithermus* WK1. Genome Biol.

[b86] Rogozin IB, Babenko VN, Wolf YI, Koonin EV, Albert VA (2005). Dollo parsimony and reconstruction of genome evolution. Parsimony, Phylogeny, and Genomics.

[b87] Farris JS (1977). Phylogenetic analysis under Dollo's law. Syst Zool.

[b88] Mirkin BG, Fenner TI, Galperin MY, Koonin EV (2003). Algorithms for computing parsimonious evolutionary scenarios for genome evolution, the last universal common ancestor and dominance of horizontal gene transfer in the evolution of prokaryotes. BMC Evol Biol.

[b89] Kjer KM, Swigonova Z, LaPolla JS, Broughton RE (2007). Why weight. Mol Phylogenet Evol.

[b90] Ames RM, Money D, Ghatge VP, Whelan S (2012). Determining the evolutionary history of gene families. Bioinformatics.

